# Supplementary material for: Tonic inhibition of the chloride/proton antiporter ClC-7 by PI(3,5)P2 is crucial for lysosomal pH maintenance
Source: eLife. 2022 Jun 7;11:e74136. doi: 10.7554/eLife.74136 (PMC9242644; doi:10.7554/eLife.74136)
Supplement: Figure 4—source data 1. [file elife-74136-fig4-data1.zip › Figure 4 Source Data 1.pdf]

Figure 4B Labeled Gels

Blot--anti PIKfyve

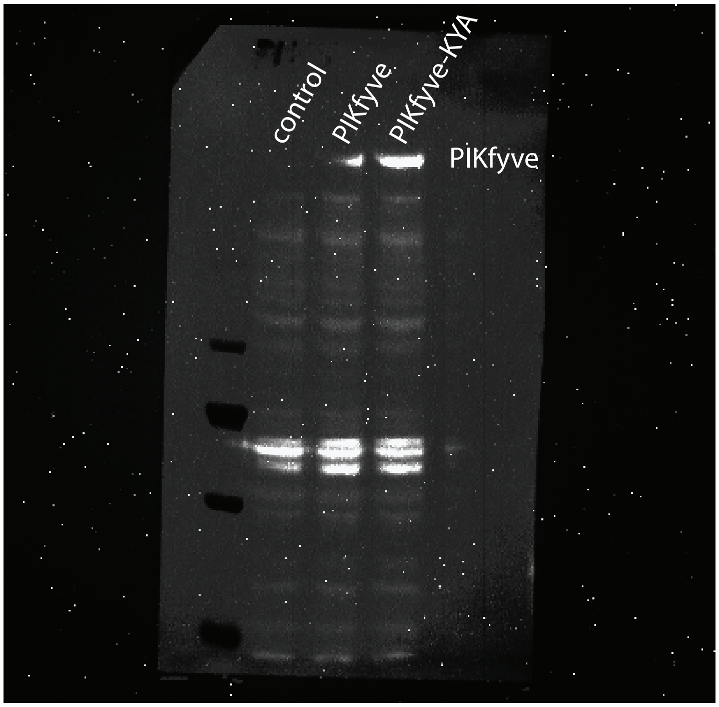

Blot--anti tubulin

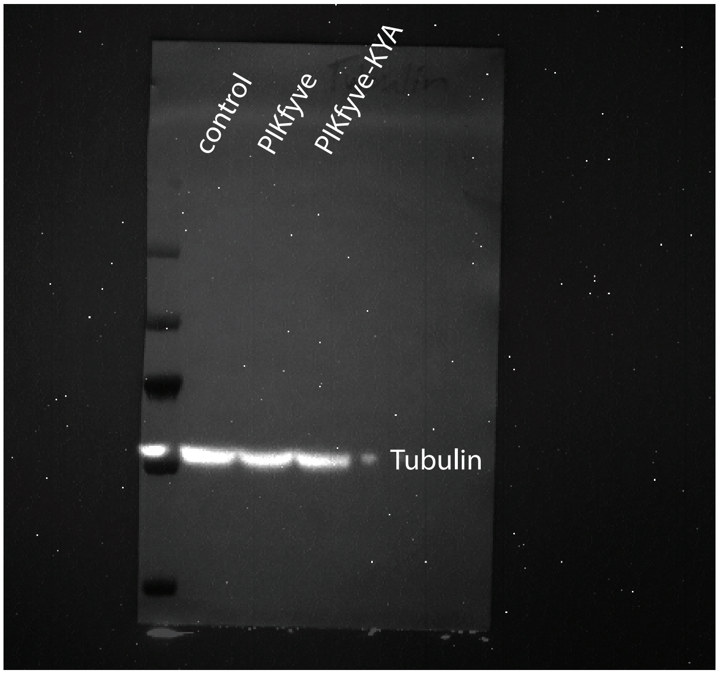

Note that these are two images of the same blot probed with different antibodies.
A photo of the blot imaged in white light (to visualize the MW standards) is superimposed on the chemiluminscent image of the same blot.
