# Supplementary figures and images for: Tonic inhibition of the chloride/proton antiporter ClC-7 by PI(3,5)P2 is crucial for lysosomal pH maintenance
Source: eLife. 2022 Jun 7;11:e74136. doi: 10.7554/eLife.74136 (PMC9242644; doi:10.7554/eLife.74136)

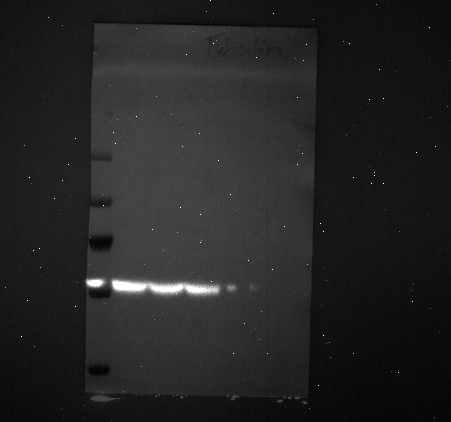

Supplement: Figure 4—source data 2. [file elife-74136-fig4-data2.zip › Figure 4 Source Data 2.png]

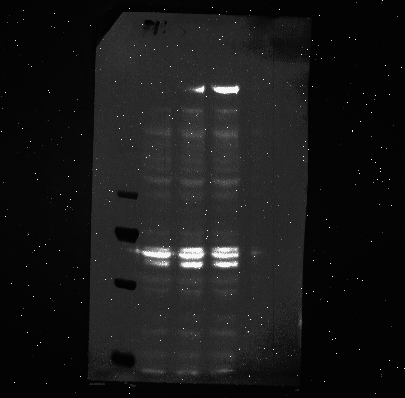

Supplement: Figure 4—source data 3. [file elife-74136-fig4-data3.zip › Figure 4 Source Data 3.png]

Figure 5A Agarose Gel of PCR product amplified from CLCN7 deletion site of WT and CLCN7 KO U2OS cells

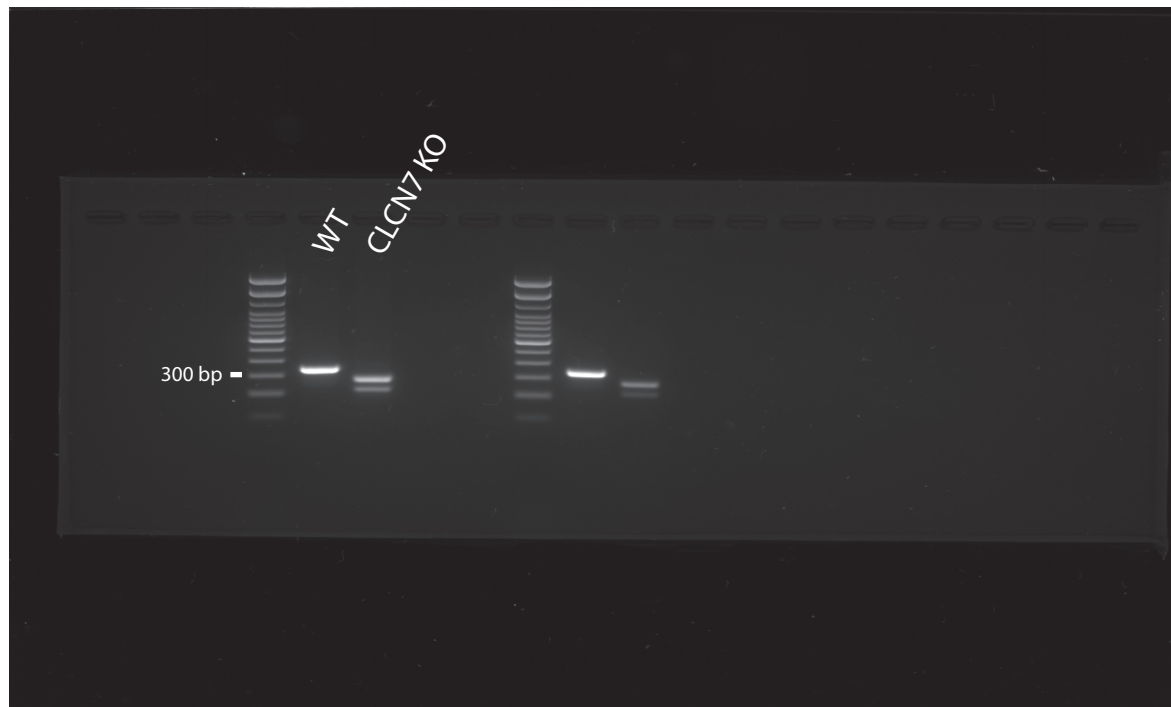

Supplement: Figure 5—source data 1. [file elife-74136-fig5-data1.zip › Figure 5 Source Data 1.pdf]

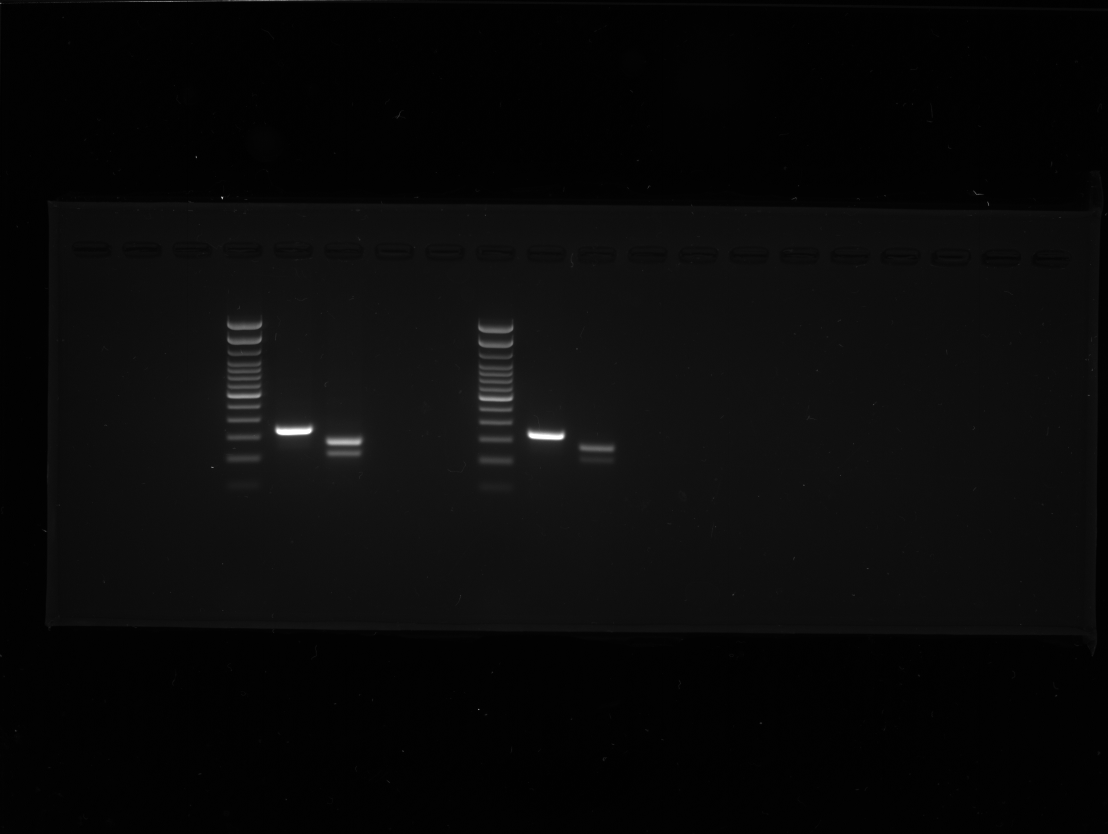

Supplement: Figure 5—source data 2. [file elife-74136-fig5-data2.zip › Figure 5 Source Data 2.png]

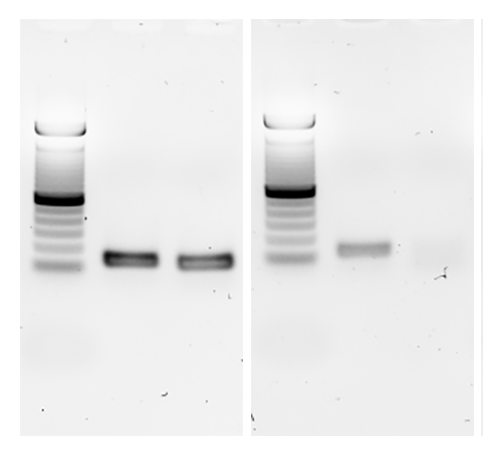

Supplement: Figure 5—figure supplement 1—source data 2. [file elife-74136-fig5-figsupp1-data2.zip › Figure 5 Supplement 1 Source Data 2.png]
