# Supplementary material for: Tonic inhibition of the chloride/proton antiporter ClC-7 by PI(3,5)P2 is crucial for lysosomal pH maintenance
Source: eLife. 2022 Jun 7;11:e74136. doi: 10.7554/eLife.74136 (PMC9242644; doi:10.7554/eLife.74136)

Figure 5 Supplement 1

Agarose Gel of RT-PCR products of GAPDH (positive control), ClC-7 WT, and ClC-7 KO U2OS cells

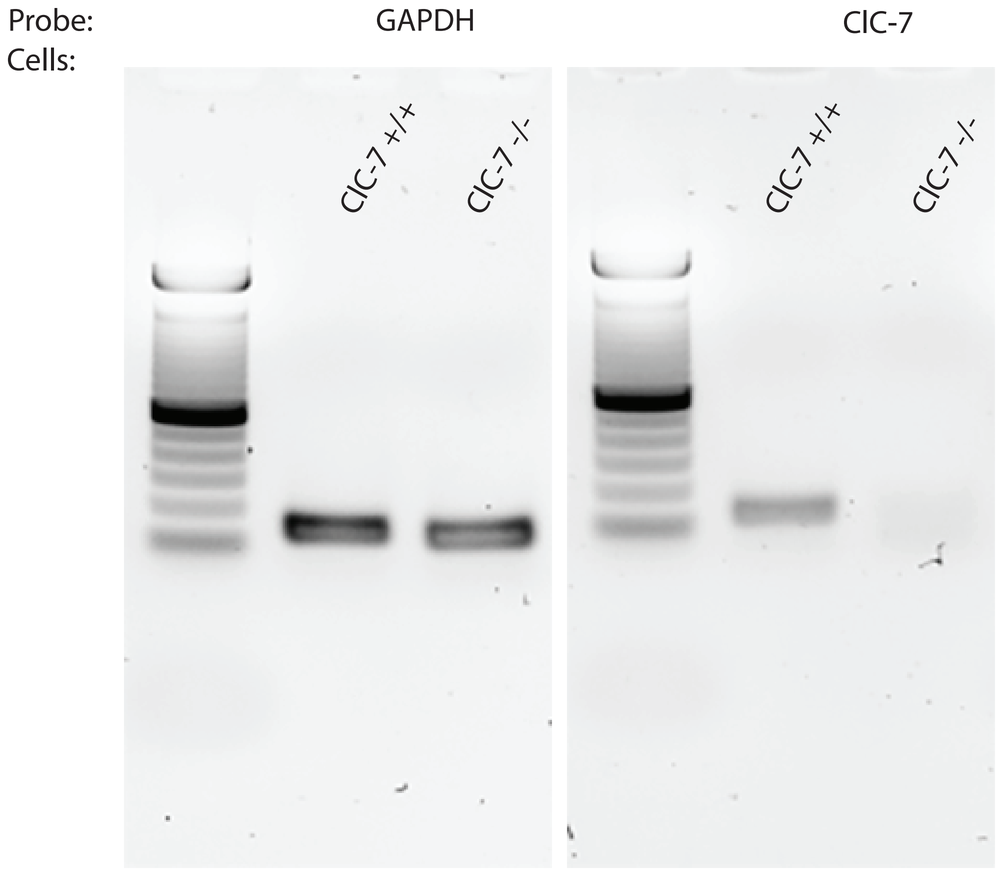

Supplement: Figure 5—figure supplement 1—source data 1. [file elife-74136-fig5-figsupp1-data1.zip › Figure 5 Supplement 1 Source Data 1.pdf]
